# Real‐world outcomes among patients with advanced or metastatic biliary tract cancers initiating second‐line treatment

**DOI:** 10.1002/cam4.5282

**Published:** 2022-10-20

**Authors:** David P. Cosgrove, Emily S. Reese, Nicole M. Fulcher, Sarah S. Bobiak, Francois‐Xavier Lamy, Arthur Allignol, Marley Boyd, Seyed H. Mahmoudpour

**Affiliations:** ^1^ Compass Oncology, The US Oncology Network Vancouver Washington USA; ^2^ EMD Serono Billerica Massachusetts USA; ^3^ Ontada The Woodlands Texas USA; ^4^ Healthcare Business of Merck KGaA Darmstadt Germany

**Keywords:** bile duct cancer, biliary cancer, chemotherapy, cholangiocarcinoma, gallbladder cancer, second line

## Abstract

**Background:**

Limited data are available regarding second‐line (2 L) treatment for advanced or metastatic biliary tract cancers (BTC) in the US real‐world setting. This study explores the rapidly evolving and growing treatment landscape in the 2 L setting for advanced or metastatic BTC with a large cohort of patients treated in a community oncology setting.

**Methods:**

Adult patients with BTC initiating 2 L treatment after a platinum‐containing first‐line between 1/1/10‐ and 6/30/19 were identified from the US Oncology Network electronic healthcare record database and followed through 12/31/19. Baseline patient and treatment characteristics were analyzed descriptively, including overall response rate (ORR) in the real‐world clinical setting. Kaplan–Meier methods were used to measure duration of response, progression‐free survival (PFS), and overall survival (OS).

**Results:**

The overall population (*N* = 160) included 74 patients (46.3%) with intrahepatic cholangiocarcinoma, 41 (25.6%) with extrahepatic cholangiocarcinoma, and 45 (28.1%) with gallbladder cancer. Thirty unique 2 L regimens were recorded for the study population, with folinic acid, fluorouracil and oxaliplatin (FOLFOX, 34.4%) and capecitabine monotherapy (20.0%) being the most common. ORR was 7.5% (95% CI, 3.9%–12.7%). From 2 L initiation, median PFS was 2.8 months (95% CI, 2.4–3.3 months), and median OS was 5.2 months (95% CI, 4.2–6.7 months).

**Conclusion:**

Results from this study provide real‐world evidence that although patients treated in the community oncology setting receive a wide variety of 2 L treatments, the regimens are consistent with those recommended by guidelines. Although responses are observed with 2 L treatment, duration is brief and associated with poor OS in patients with advanced or metastatic disease.

## 1 INTRODUCTION

Biliary tract cancer (BTC) represents a heterogeneous group of cancers associated with poor outcomes that includes intrahepatic cholangiocarcinoma (ICCA), extrahepatic cholangiocarcinoma (ECCA), gallbladder cancer, and ampulla of vater cancers. The American Cancer Society estimates that in the United States in 2022, 12,130 patients will be newly diagnosed with BTC, and there will be 4400 BTC–related deaths.^1^ In the Western world, the incidence of BTC is between 0.35 and 2 per 100,000 annually.^2^ The estimated 5‐year survival rates among patients diagnosed with BTC range from 2%–70% for gallbladder cancer depending on stage, 2% to 30% for ECCA, and 2%–15% for ICCA.^3^ Gallbladder cancer is the most frequent BTC, comprising 80%–90% of all BTCs and representing 10%–25% of primary hepatic malignancies in the world, and ICCA and ECCA are the second and third most common BTC.^3^ Between 1999 and 2013, rates of ICCA and ECCA in the US consistently increased in all sex and racial/ethnic groups.^4^

Curative treatments, including surgery and adjuvant therapy, are common for early‐stage BTC; however, most patients are diagnosed with later‐stage disease. Systemic chemotherapy is the standard approach for the initial treatment of patients with unresectable locally advanced or metastatic BTC. Based on the landmark phase 3 ABC‐02 trial,^5^ combination therapy with gemcitabine and cisplatin is the standard first‐line (1 L) treatment for advanced BTC, although this regimen can be associated with severe toxicities, including renal and neurological toxicity, ototoxicity, and severe myelosuppression.^5^, ^6^

No global standard of care (SOC) exists in second line (2 L), although National Comprehensive Cancer Network (NCCN) guidelines now include FOLFOX (folinic acid, fluorouracil and oxaliplatin) as the preferred 2 L therapy based on findings from the phase 3, open‐label, randomized, multicenter trial ABC‐06.^7^ This trial compared active symptom control (ASC) + FOLFOX vs ASC alone in the 2 L setting and found that in patients progressing after 1 L gemcitabine +1cisplatin, the addition of FOLFOX to ASC was associated with improved overall survival (OS; HR, 0.69; *p* = 0.031), with a benefit of 6.2 versus 5.3 months. Recent developments in the treatment landscape for advanced BTC have expanded the available treatments to include targeted therapies and immunotherapy agents.^6^ This study provided the first level 1 evidence for 2 L treatment after SOC 1 L therapy.^7^

To date, information is sparse regarding 2 L treatment for advanced or metastatic BTC in the US real‐world setting. Existing studies report findings outside of the US, report data only from academic centers, include only older populations, or use older data.^8^, ^9^, ^10^, ^11^, ^12^, ^13^, ^14^, ^15^, ^16^, ^17^, ^18^, ^19^ Therefore, with its large cohort of patients treated in the community oncology setting, our study aimed to provide real‐world insight into the rapidly evolving and growing 2 L treatment landscape for advanced or metastatic BTC.

## 2 MATERIALS AND METHODS

### 2.1 Study overview, data, and patient selection

This was a retrospective observational cohort study. Patient profiles, treatment patterns, and selected outcomes were assessed for patients with advanced or metastatic BTC who received care in The US Oncology Network. All patients received systemic treatment following a platinum‐based 1 L regimen between January 1, 2010, and June 30, 2019. Patients were followed longitudinally until the last patient record, date of death, or end of study period (December 31, 2019), whichever occurred first. The US Oncology Network is affiliated with approximately 1400 physicians in >480 sites across 40 states in the US, representing approximately 12% of US patients newly diagnosed with cancer during the analysis period.^20^ Most study data originated from the electronic health record (EHR) database of The US Oncology Network, iKnowMed (iKM). The Social Security Administration's Limited Access Death Master File was used as a supplemental source of death records.

For inclusion, patients were required to have a confirmed diagnosis of advanced or metastatic BTC, be aged ≥18 years at diagnosis, and have initiated a 2 L systemic anticancer treatment after a platinum‐containing 1 L treatment. Patients who initiated treatment for BTC between January 1, 2010 and June 30, 2019 were eligible. Patients had to have ≥1 additional visit in The US Oncology Network following advanced or metastatic BTC diagnosis. Patients were excluded if they were enrolled in interventional clinical trials or diagnosed with other primary cancers during the 3 years before 1 L treatment. During examination of unstructured data, certain critical eligibility criteria were confirmed, including prior platinum therapy, diagnosis of advanced or metastatic BTC, and presence of baseline measurable disease for the purpose of achieving study objectives related to treatment response. When a patient was found to be eligible based on structured data but ineligible based on confirmation of key criteria during the chart review, the patient was disqualified from the study.

### 2.2 Study end points and statistical analysis

This study had several objectives. First, patient and treatment characteristics were identified. Second, the overall response rate (ORR) achieved with 2 L chemotherapy was evaluated using Response Evaluation Criteria In Solid Tumors version 1.1 (RECIST 1.1) as a guide^21^ together with physician‐assessed response. Third, duration of response (DOR), progression‐free survival (PFS), and OS for 2 L treatment were all measured. The incidence of toxicities considered to be severe was also assessed; ie, the toxicities associated with treatment discontinuation, hospitalization, emergency department visits, or death.

Descriptive analyses were conducted to evaluate the demographic, clinical, and treatment characteristics of the study population. Kaplan–Meier methods were used to assess DOR, OS, and PFS. DOR in 2 L was defined as time (months) from complete response (CR) or partial response (PR) during 2 L treatment as guided by the composite measure of response until progression of disease (PD) or death. OS was defined as time (months) between initiation of 2 L treatment and date of death (any cause). PFS was defined as time (months) from initiation of 2 L treatment until the earliest of PD (using a composite measure of physician‐assessed responses and response as guided by RECIST 1.1) or death. The incidence of severe toxicities during 2 L treatment was reported per 100 person‐years (PYs).

Concordance of the physician‐assessed response and RECIST 1.1–guided objective response was evaluated among patients with both types of assessments. The proportions of concordant and discordant response categories as well as Cohen κ (weighted and unweighted) were calculated to describe the degree of agreement between scan report–based RECIST‐like response and physician‐reported response abstracted using all four response categories (i.e., CR, Response ‐not otherwise specified [R‐NOS], Stable Disease [SD], PD) The physician‐reported PR category included R‐NOS and mixed response if the patient continued treatment.

## 3 RESULTS

### 3.1 Study population

Across all US Oncology Network clinics, 5211 adult patients were identified with a diagnosis of BTC (see Figure S1). Using structured data fields, 2759 patients were excluded because they did not initiate a qualifying treatment within The US Oncology Network during the study identification period. Additional reasons for exclusion included <2 office visits following diagnosis (*n* = 33), diagnosis with another primary cancer or received treatment for other primary cancer (*n* = 298), and clinical trial participation (*n* = 88). An additional 1626 patients were excluded for not initiating a 2 L systemic treatment following a 1 L platinum‐based therapy. A total of 160 patients from a group of 361 randomly selected patients met the study inclusion criteria. The population included 74 patients (46.3%) with ICCA, 41 (25.6%) with ECCA, and 45 (28.1%) with gallbladder cancer.

### 3.2 Patient characteristics

Patient demographic and clinical characteristics are presented in Table 1 and Table S1. Overall, the median age was 65 years, and 52.5% of patients were female. The majority of patients (66.3% [n = 106]) had an Eastern Cooperative Oncology Group performance status of 0 or 1 at the start of 2 L therapy. At initial BTC diagnosis, most patients (82.5% [*n* = 132]) had stage III/IV disease. Median follow‐up time was 4.5 months (range, 0.5–43.6 months).

**TABLE 1:** Baseline demographic and clinical characteristics

Analysis variable	Overall	BTC subtype		
		ICCA	ECCA	Gallbladder
Patients, *n*	160	74	41	45
Age at baseline, years				
Median (min, max)	65 (36, 86)	65 (36, 86)	65 (37, 84)	64 (44, 78)
Sex, *n* (%)				
Female	84 (52.5)	34 (45.9)	18 (43.9)	32 (71.1)
Race, *n* (%)				
White	114 (71.3)	53 (71.6)	32 (78.0)	29 (64.4)
Not documented	27 (16.9)	15 (20.3)	3 (7.3)	9 (20.0)
Other	19 (11.9)	6 (8.1)	6 (14.6)	7 (15.6)
Smoking history, *n* (%)				
Current/former	70 (6.3)	37 (9.5)	18 (4.9)	15 (2.2)
Never	69 (43.1)	28 (37.8)	16 (39.0)	25 (55.6)
Not documented	21 (13.1)	9 (12.2)	7 (17.1)	5 (11.1)
Practice region, *n* (%)				
West	56 (35.0)	22 (29.7)	19 (46.3)	15 (33.3)
Midwest	51 (31.9)	27 (36.5)	11 (26.8)	13 (28.9)
South	39 (24.4)	17 (23.0)	7 (17.1)	15 (33.3)
Northeast	14 (8.8)	8 (10.8)	4 (9.8)	2 (4.4)
ECOG PS at baseline, *n* (%)				
0	17 (10.6)	5 (6.8)	5 (12.2)	7 (15.6)
1	89 (55.6)	41 (55.4)	26 (63.4)	22 (48.9)
2	27 (16.9)	16 (21.6)	6 (14.6)	5 (11.1)
3	1 (0.6)	0	1 (2.4)	0
Not documented	26 (16.3)	12 (16.2)	3 (7.3)	11 (24.4)
BMI group at baseline, *n* (%)				
Underweight (<18.5 kg/m^2^)	3 (1.9)	2 (2.7)	0	1 (2.2)
Normal (18.5 to <25.0 kg/m^2^)	53 (33.1)	22 (29.7)	16 (39.0)	15 (33.3)
Overweight (25.0 to <30.0 kg/m^2^)	52 (32.5)	23 (31.1)	12 (29.3)	17 (37.8)
Obese (≥30 kg/m^2^)	51 (31.9)	26 (35.1)	13 (31.7)	12 (26.7)
Not documented	1 (0.6)	1 (1.4)	0	0
Stage at initial BTC diagnosis, *n* (%)				
IA	2 (1.3)	1 (1.4)	1 (2.4)	0
IB	1 (0.6)	0	1 (2.4)	0
II	6 (3.8)	5 (6.8)	0	1 (2.2)
IIA	2 (1.3)	0	1 (2.4)	1 (2.2)
IIB	3 (1.9)	0	2 (4.9)	1 (2.2)
IIIA	8 (5.0)	2 (2.7)	4 (9.8)	2 (4.4)
IIIB	5 (3.1)	1 (1.4)	2 (4.9)	2 (4.4)
IIIC	1 (0.6)	1 (1.4)	0	0
IV	52 (32.5)	20 (27.0)	18 (43.9)	14 (31.1)
IVA	9 (5.6)	5 (6.8)	2 (4.9)	2 (4.4)
IVB	57 (35.6)	33 (44.6)	6 (14.6)	18 (40.0)
Not documented	14 (8.8)	6 (8.1)	4 (9.8)	4 (8.9)
Metastatic sites recorded up to 2 L initiation, *n* (%)				
Liver	106 (66.3)	57 (77.0)	22 (53.7)	27 (60.0)
Lymph node	69 (43.1)	32 (43.2)	19 (46.3)	18 (40.0)
Lung	39 (24.4)	17 (23.0)	13 (31.7)	9 (20.0)
Peritoneal	23 (14.4)	8 (10.8)	6 (14.6)	9 (20.0)
Bone	21 (13.1)	17 (23.0)	3 (7.3)	1 (2.2)
Other	17 (10.6)	4 (5.4)	3 (7.3)	10 (22.2)
Adrenal gland	2 (1.3)	2 (2.7)	0	0
Albumin result, *n* (%)				
Low (<3.5 g/dL)	52 (32.5)	26 (35.1)	17 (41.5)	9 (20.0)
Normal (3.5–5 g/dL)	104 (65.0)	47 (63.5)	23 (56.1)	34 (75.6)
Not documented	4 (2.5)	1 (1.4)	1 (2.4)	2 (4.4)
Bilirubin, *n* (%)				
Normal (0.1–1 mg/dL)	139 (86.9)	66 (89.2)	35 (85.4)	38 (84.4)
Elevated (>1 mg/dL)	17 (10.6)	7 (9.5)	4 (9.8)	6 (13.3)
Not documented	4 (2.5)	1 (1.4)	2 (4.9)	1 (2.2)
CA 19–9 result, *n* (%)				
Normal (0–37 μ/mL)	29 (18.1)	11 (14.9)	5 (12.2)	13 (28.9)
Elevated (>37 μ/mL)	110 (68.8)	51 (68.9)	31 (75.6)	28 (62.2)
Not documented	21 (13.1)	12 (16.2)	5 (12.2)	4 (8.9)
CEA result, *n* (%)				
Normal (0–2.5 ng/dL)	31 (19.4)	16 (21.6)	3 (7.3)	12 (26.7)
Elevated (>2.5 ng/dL)	56 (35.0)	22 (29.7)	16 (39.0)	18 (40.0)
Not documented	73 (45.6)	36 (48.6)	22 (53.7)	15 (33.3)
Microsatellite instability status within 1 year of 2 L initiation, *n* (%)				
Indeterminate	2 (1.3)	1 (1.4)	1 (2.4)	0
Stable	21 (13.1)	12 (16.2)	2 (4.9)	7 (15.6)
Not documented	137 (85.6)	61 (82.4)	38 (92.7)	38 (84.4)
INR, *n* (%)				
Not documented	72 (45.0)	35 (47.3)	13 (31.7)	24 (53.3)
Normal (0.8–1.1)	54 (33.8)	21 (28.4)	17 (41.5)	16 (35.6)
Elevated (>1.1)	34 (21.3)	18 (24.3)	11 (26.8)	5 (11.1)
PD‐L1 result, *n* (%)				
Negative	18 (11.3)	10 (13.5)	3 (7.3)	5 (11.1)
Positive	4 (2.5)	2 (2.7)	0	2 (4.4)
Not documented	138 (86.3)	62 (83.8)	38 (92.7)	38 (84.4)
Follow‐up time from 2 L initiation, months				
Mean (SD)	6.6 (6.7)	6.1 (5.9)	7.5 (7.8)	6.6 (7.1)
Median (min, max)	4.5 (0.5, 43.6)	4.1 (0.6, 39.8)	4.0 (0.5, 35.8)	4.8 (0.5, 43.6)

Abbreviations: 2 L, second line; BMI, body mass index; BTC, biliary tract cancer; CA 19–9, carbohydrate antigen 19–9; CEA, carcinoma embryonic antigen; ECCA, extrahepatic cholangiocarcinoma; ECOG PS, Eastern Cooperative Oncology Group performance status; ICCA, intrahepatic cholangiocarcinoma; INR, international normalized ratio; max, maximum; min, minimum; PD‐L1, programmed death ligand 1.

### 3.3 Treatment characteristics

The median time from the advanced or metastatic BTC diagnosis to initiation of 2 L therapy was 5.7 months (range, 1.3–21.4 months). The majority of patients (93.8%) received gemcitabine in combination with cisplatin or carboplatin as their 1 L regimen, with a median treatment duration of 3.5 months (range, 0.6–20.5 months). There was wide variability in 2 L treatments: Thirty unique regimens were recorded for the study population. FOLFOX (34.4%) and capecitabine monotherapy (20.0%) were the most common 2 L therapies. Gemcitabine in combination with cisplatin or carboplatin was used in only 3.1% of regimens (*n* = 5), while 2.5% (*n* = 4) of the study regimens included immunotherapy (pembrolizumab or nivolumab). Median treatment duration for 2 L regimens was 1.9 months (range, 0.0–11.7 months). Forty‐six patients (28.8%) received a third‐line treatment (Table 2).

**TABLE 2:** Treatment characteristics

Analysis variable	Overall	BTC subtype		
		ICCA	ECCA	Gallbladder carcinoma
Time from initial BTC diagnosis to 2 L initiation, months				
Patients with available data, *n*	160	74	41	45
Mean (SD)	8.3 (7.5)	8.1 (8.2)	9.3 (6.3)	7.7 (7.5)
Median (min, max)	6.3 (1.8, 62.6)	6.1 (1.8, 62.6)	7.6 (2.2, 38.1)	5.6 (2.2, 38.2)
Time from documented advanced/metastatic disease diagnosis to 2 L initiation, months				
Patients with available data, *n*	160	74	41	45
Mean (SD)	6.7 (4.3)	7.1 (5.1)	7.0 (3.6)	5.8 (3.3)
Median (min, max)	5.7 (1.3, 21.4)	5.8 (1.3, 21.4)	6.4 (2.2, 17.7)	5.2 (1.4, 16.6)
Duration of 1 L treatment, months				
Mean(SD)	4.3 (3.5)	4.3 (3.9)	4.7 (3.6)	3.8 (2.7)
Median (min, max)	3.5 (0.6, 20.5)	3.0 (0.6, 20.5)	3.7 (1.0, 15.9)	3.6 (0.7, 14.9)
1 L regimens, *n* (%)				
Gemcitabine + cisplatin	140 (87.5)	62 (83.8)	40 (97.6)	38 (84.4)
Gemcitabine + carboplatin	10 (6.3)	7 (9.5)	1 (2.4)	2 (4.4)
Other platinum containing	10 (6.3)	5 (6.8)	0	5 (11.1)
Duration of 2 L treatment, months				
Mean (SD)	2.5 (2.0)	2.7 (2.0)	2.2 (2.3)	2.3 (1.6)
Median (min, max)	1.9 (0.0, 11.7)	2.1 (0.0, 8.9)	1.4 (0.3, 11.7)	1.9 (0.2, 7.2)
2 L regimens, *n* (%)				
Fluorouracil + leucovorin + oxaliplatin (FOLFOX)	55 (34.4)	26 (35.1)	12 (29.3)	17 (37.8)
Capecitabine	32 (20.0)	14 (18.9)	8 (19.5)	10 (22.2)
Gemcitabine + capecitabine	15 (9.4)	6 (8.1)	5 (12.2)	4 (8.9)
Capecitabine + oxaliplatin	15 (9.4)	8 (10.8)	4 (9.8)	3 (6.7)
Fluorouracil + oxaliplatin	5 (3.1)	2 (2.7)	2 (4.9)	1 (2.2)
Gemcitabine + cisplatin	4 (2.5)	3 (4.1)	0	1 (2.2)
Fluorouracil + irinotecan + leucovorin (FOLFIRI)	4 (2.5)	1 (1.4)	2 (4.9)	1 (2.2)
Immunotherapy‐based (nivolumab or pembrolizumab containing)	4 (2.5)	2 (2.7)	1 (2.4)	1 (2.2)
Other regimens	26 (16.3)	12 (16.2)	7 (17.1)	7 (15.6)

Abbreviations: 1 L, first line; 2 L, second line; BTC, biliary tract cancer; ECCA, extrahepatic cholangiocarcinoma; ICCA, intrahepatic cholangiocarcinoma; max, maximum; min, minimum.

### 3.4 Response to 2 L Treatment

No patients achieved a CR to 2 L treatment, although 12 achieved either PR (RECIST like) or R‐NOS (physician assessed). In the overall population, the ORR was 7.5% (95% CI, 3.9%–12.7% [*n* = 12]). Median DOR to 2 L treatment was not reached during the limited follow‐up (median follow‐up, 4.5 months; 3 events); however, 53.3% of 2 L responders had responses lasting ≥6 months (Table 3 and Figure S2).

**TABLE 3:** Summary of response to second‐line treatment

Response	Overall	BTC subtype		
		ICCA	ECCA	Gallbladder carcinoma
Total patient count, *n*	160	74	41	45
Overall response rate (ORR) including all patients (95% CI), %	7.5 (3.9–12.7)	6.8 (2.2–15.1)	9.8 (2.7–23.1)	6.7 (1.4–18.3)
Duration of response, median (95% CI), months	NR (1.3‐NR)	NR (1.3‐NR)	NR (NR‐NR)	2.5 (1.4–3.5)
Duration of response >6 months (95% CI), %	53.3 (8.5–85.2)	75.0 (12.8–96.1)	NA	NA
Best overall response, *n* (%)				
Complete response	0	0	0	0
Partial response	8 (5.0)	4 (5.4)	4 (9.8)	0
Response (NOS)	4 (2.5)	1 (1.4)	0	3 (6.7)
Stable disease	60 (37.5)	31 (41.9)	14 (34.1)	15 (33.3)
Progressive disease	55 (34.4)	19 (25.7)	16 (39.0)	20 (44.4)
Mixed response	1 (0.6)	0	0	1 (2.2)
Not evaluated	32 (20.0)	19 (25.7)	7 (17.1)	6 (13.3)

*Note*: The composite response measurement was determined by a hierarchy of RECIST‐like and physician‐assessed responses. Hierarchy: (1) RECIST‐like, where all baseline target lesions had follow‐up imaging; (2) RECIST‐like, where some baseline target lesions had follow‐up imaging; (3) physician assessed, where imaging was noted; and (4) physician assessed, where imaging was not noted.

Abbreviations: ECCA, extrahepatic cholangiocarcinoma; ICCA, intrahepatic cholangiocarcinoma; NOS, not otherwise specified; NR, not reached; RECIST, response evaluation criteria in solid tumors.

Tumor responses among the 73 patients (5.6%) with both physician‐assessed and RECIST‐like responses are presented in Table 4. More physician‐assessed responses than RECIST‐like responses were categorized as CRs or PRs (20 [27.4%] vs. 6 [8.2%]). Although there seemed to be an agreement on responses (PR or CR), most RECIST‐like SDs were recognized as PR or PD by physicians. There were 46 RECIST‐like SDs (63.0%) and only 12 according to physician assessment (16.4%). Of the remaining physician‐assessed PRs, all 14 were classified as SD by RECIST‐like criteria. The κ coefficient was 0.25 (95% CI, 0.12–0.38), and the weighted κ was 0.37 (95% CI, 0.24–0.50), indicating low concordance between physician‐assessed and RECIST‐like responses.

**TABLE 4:** Treatment response by assessment method

	Physician‐assessed^a^				
	Complete response, *n* (%)	Partial response, *n* (%)^b^	Stable disease, *n* (%)	Progressive disease, *n* (%)	Total, *N*
RECIST‐like^a^					
Complete response, *n* (%)	0	0	0	0	**0**
Partial response, *n* (%)	1 (100)	5 (26.3)	0	0	**6**
Stable disease, *n* (%)	0	14 (73.7)	10 (83.3)	22 (53.7)	**46**
Progressive disease, *n* (%)	0	0	2 (16.7)	19 (46.3)	**21**
Total, *N*	**1**	**19**	**12**	**41**	**73**

*Note*: κ = 0.2512 (95% CI, 0.1238–0.3785), Weighted k = 0.3722 (95% CI, 0.2400–0.5044).

Abbreviation: RECIST, response evaluation criteria in solid tumors.

^a^ Only includes patients evaluated using both methods.

^b^ Includes physician‐assessed responses categorized as response not otherwise specified (R‐NOS). Physicianassessed mixed responses are not included in this analysis.

**FIGURE 1:**
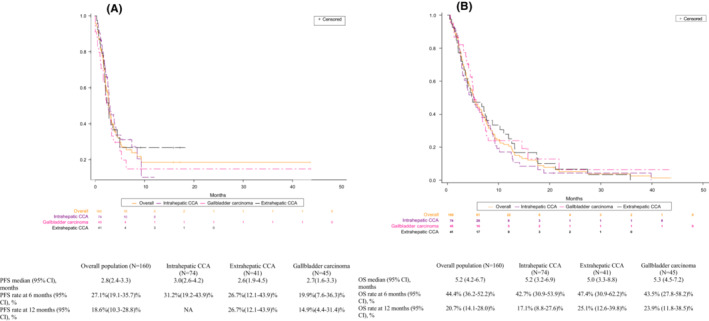
(A) Kaplan–Meier curve and estimates of progression‐free survival from initiation of second‐line treatment and (B) Kaplan–Meier curve and estimates of overall survival from initiation of second‐line treatment.

Using the composite measure of RECIST‐like and physician‐assessed responses, median PFS from the initiation of 2 L treatment was 2.8 months (95% CI, 2.4–3.3 months) in all patients with BTC and 3.0 months (95% CI, 2.6–4.2 months), 2.6 months (95% CI, 1.9–4.5 months), and 2.7 months (95% CI, 1.6–3.3 months) in patients with ICCA, ECCA, and gallbladder cancer, respectively (Figure 1A). Overall, the PFS rate at 6 months was 27.1% (95% CI, 19.1%–35.7%); it was highest in patients with ICCA at 31.2% (95% CI, 19.2%–43.9%), followed by ECCA at 26.7% (95% CI, 12.1%–43.9%) and gallbladder cancer at 19.9% (95% CI, 7.6%–36.3%).

At the time of study analysis, 131 (81.9%) of the 160 patients had records of death. Median OS from initiation of 2 L treatment was 5.2 months (95% CI, 4.2–6.7 months) among all study patients (Figure 1B). OS was consistent among subtypes. In the subsets of patients with ICCA, ECCA, and gallbladder cancer, median OS was 5.2 months (95% CI, 3.2–6.9 months), 5.0 months (95% CI, 3.3–8.8 months), and 5.3 months (95% CI, 4.5–7.2 months), respectively. Twelve‐month survival was 20.7% (95% CI, 14.1%–28.0%) overall; in patients with ICCA, ECCA, and gallbladder cancer, it was 17.1% (95% CI, 8.8%–27.6%), 25.1% (95% CI, 12.6%–39.8%), and 23.9% (95% CI, 11.8%–38.5%), respectively.

### 3.5 Toxicity in 2 L

All patients were examined for toxicity during 2 L treatment. Severe toxicity resulting in treatment discontinuation, hospitalization, emergency department visit, or death was recorded for 20 patients (12.5%). Severe toxicity incidence rates are described in Table 5. Among patients at risk for developing toxicities, fatigue had the highest incidence rate (33.3/100 PYs), followed by diarrhea (24.9/100 PYs) and infusion reactions (15.4/100 PYs).

**TABLE 5:** Severe toxicity incidence during 2 L treatment

Severe toxicity	Patients at risk, n*	Patients with events, *n*	Incidence per 100 person‐years
Acute kidney injury	158	2	6.1
Diarrhea	152	8	24.9
Fatigue	149	11	33.3
Infection	159	1	3.0
Infusion reactions	155	5	15.4

*Note*: 2 L, second line.

* Incidence is calculated among patients at risk for developing the toxicity. At‐risk patients are those with no evidence of toxicity during the 2 L baseline period.

## 4 DISCUSSION

Documentation of real‐world outcomes associated with treatment in advanced or metastatic BTC is limited in the US.^9^, ^13^, ^14^, ^15^, ^19^ Therefore, the aim of this study was to contribute to literature on the real‐world 2 L treatments and outcomes of patients in BTC using historical data. Using The US Oncology Network/Ontada EHR database and medical charts to assess the patient and treatment characteristics, response to chemotherapy, and clinical outcomes in patients with advanced or metastatic BTC who received 2 L systemic treatment following a 1 L platinum‐based treatment, this study provides real‐world clinical data representing a multisite, heterogeneous sample of patients across the US treated in the community oncology setting.

In this study, patients received a wide variety of 2 L treatments, including 30 unique regimens. During the time period of the study (2010–2019), over half of patients (54.4%) used either FOLFOX (NCCN‐preferred regimen) or capecitabine monotherapy. Regimens of gemcitabine + cisplatin or carboplatin, which are SOC in 1 L, were used sparingly as 2 L treatments (3.1%). In a retrospective study of National Cancer Institute Surveillance, Epidemiology, and End Results (SEER) cancer registry data linked with Medicare enrollment and claims data from 2010 to 2014, Danese et al also reported wide variability in 2 L treatments among patients with stage III/IV BTC disease, which included 25 unique combinations.^19^ The most common 2 L regimens in the SEER‐Medicare data included gemcitabine monotherapy (19.5%), capecitabine monotherapy (12.7%), FOLFOX (12.3%), and gemcitabine + cisplatin (11.4%).^19^ In a separate study during 2010–2015, Lowery et al reported that >60% of 2 L patients used 5‐fluorouracil–based regimens and 18% used gemcitabine‐based therapies.^13^ The regimens used in this study, which included later data through 2019, demonstrated 2 L regimen use more closely aligned with current NCCN guidelines, which recommend FOLFOX as the preferred regimen in treatment after BTC progresses.^6^

In the advanced or metastatic BTC population, no patients achieved a CR during 2 L treatment. A PR (or R‐NOS) was reported in only 12 patients, indicating a low ORR of 7.5%, which is similar to the ORR reported in ABC‐06 (5%).^7^ The median DOR among these patients was not reached during the study period; 53% of them did not have an event at 6 months, although only 3 events were captured among responders. In the overall population, PFS was <3 months and OS approximately 5.2 months. Both durations are approximately 1 month shorter than results reported in ABC‐06, which may reflect a population with more severe disease in this real‐world setting. Real‐world DOR in the 2 L setting has not previously been reported.

This study's results are consistent with those of a retrospective study of patients with advanced or metastatic BTC who received 2 L systemic treatment at 1 of 17 French hospitals and treatment centers from November 2002 to December 2013 after 1 L treatment with gemcitabine + platinum failed. Over a median follow‐up of 26 months, Brieau et al reported an objective response rate to 2 L treatment of 11.8%.^8^ Results from our study are also consistent with the ABC‐06 phase 3 clinical trial among 162 adult patients in the UK with advanced or metastatic BTC initiating ASC + FOLFOX after disease progression with 1 L gemcitabine + cisplatin. In the ABC‐06 trial, an objective response was observed in 4 (5%) of the 81 patients initiating ASC + FOLFOX.^7^

Measures of PFS in this study are similar to those of several other studies, which have reported PFS from initiation of 2 L treatment ranging from 2.4 to 4.0 months.^7^, ^8^, ^10^, ^14^, ^15^, ^16^ In the ABC‐06 clinical trial, Lamarca et al reported a median PFS of 4.0 months (95% CI, 3.2–5.0 months) from the start of 2 L treatment.^7^ Brieau et al reported a median PFS of 3.2 months (95% CI, 2.8–4.0 months) from the start of 2 L.^8^ In addition, the PFS results reported here are in line with a retrospective study of 198 patients with stage III/IV BTC treated at three US institutions during the period from April 2010 to March 2015. In this real‐world study, time to disease progression, measured by time to treatment failure from start of 2 L, was 2.2 months (95% CI, 1.8–2.7 months).^13^

As with findings on disease progression, 2 L OS results from our analyses are consistent with those reported in the literature. Although the median OS of 11 months reported by Lowery et al^13^ and median OS of 17 months reported in a recent multicenter, population‐based cohort study by Zaidi et al^18^ are somewhat longer than the 6.6 to 7.7‐month median OS reported by others and nearly 2 to 3 times the 5.2‐month median OS of our study, our finding is in line with other studies.^7^, ^8^, ^9^, ^10^, ^14^, ^16^, ^17^ Recent findings presented for the ABC‐06 trial describe a median OS of 6.2 months (95% CI, 5.4–7.6 months) from the start of 2 L treatment in patients treated with ASC + FOLFOX.^7^ Similarly, in their retrospective examination of Medicare patients using SEER data from 2010 to 2014, Bobiak et al reported a median OS of 5.6 months (95% CI, 4.6–6.5 months) from the start of 2 L treatment in 220 patients.^9^

### 4.1 Limitations

The results of this study should be considered in the context of the strengths and weaknesses of its design. As a retrospective observational study, treatment selection was at the discretion of providers’ clinical consideration, and underlying patient differences may have contributed to observed variation across groups, particularly for clinical outcomes.

Study data were sourced from iKM, which was originally populated for clinical practice purposes, not research. Consequently, as with all administrative database research, data entry errors or missing values may have occurred and introduced some level of misclassification bias.

This analysis used a composite measure of response that included RECIST‐guided assessments and physician assessments. In real‐world clinical practice, treatment responses usually are not recorded in a manner consistent with RECIST responses in clinical trial research. Physician‐assessed responses are typically more subjective than RECIST‐based assessments and may include non‐RECIST symptomatic criteria. Further, the timing of repeat imaging used in RECIST‐like responses may vary across physicians, practices, and insurers whereas traditional RECIST‐based assessment is determined based on quantitative measurements of target lesions at specific, predefined intervals (e.g., every 12 weeks).^20^ In our study, the time between the baseline imaging report and the subsequent imaging ranged from 1 to 27 weeks.

Data for this study were examined for patients initiating 2 L treatment between 1/1/10 and 6/30/19 with follow‐up through 12/31/19. Recent developments in the treatment of advanced BTC, including the approval and use of recently approved targeted therapies pemigatinib and ivosidenib are not captured in this study.^6^

The results of this study may not be generalizable to community oncology clinics outside The US Oncology Network or those in The US Oncology Network that do not utilize the full EHR capabilities. The US Oncology Network clinics that use full EHR capabilities may have different patient populations and physician prescribing practices due to the decision‐support tools of the EHR, such as Value Pathways. Also, The US Oncology Network clinics are located throughout the US, and regional practice differences may have influenced the results.

### 4.2 Conclusions

BTC is an aggressive disease that traditionally has lacked standard therapeutic options in the 2 L setting. To date, limited data exist regarding treatment characteristics and response in the real‐world setting. Results from this study provide real‐world evidence that although patients treated in the community oncology setting receive a wide variety of 2 L treatments, the regimens are consistent with those recommended by guidelines. We believe this is the first study to report data on DOR in a real‐world, 2 L setting. These results indicate that although responses are observed with 2 L treatment, duration is brief and associated with poor OS in patients with advanced or metastatic disease. These results underscore the need for novel therapeutic approaches in the 2 L setting. Future studies examining recent therapeutic approaches with targeted treatments and immunotherapies may be warranted.

## AUTHOR CONTRIBUTIONS

**David Cosgrove:** Conceptualization (equal); methodology (equal); supervision (equal); validation (equal); writing – original draft (equal); writing – review and editing (equal). **Emily Reese:** Conceptualization (equal); investigation (equal); methodology (equal); project administration (equal); writing – original draft (equal); writing – review and editing (equal). **Nicole Fulcher:** Conceptualization (equal); data curation (equal); formal analysis (equal); investigation (equal); methodology (equal); project administration (equal); resources (equal); validation (equal); writing – original draft (equal); writing – review and editing (equal). **Sarah Bobiak:** Conceptualization (equal); investigation (equal); methodology (equal); project administration (equal); writing – review and editing (equal). **Francois‐Xavier Lamy:** Conceptualization (equal); investigation (equal); methodology (equal); project administration (equal); resources (equal); supervision (equal); writing – review and editing (equal). **Arthur Allignol:** Formal analysis (equal); funding acquisition (lead); investigation (equal); methodology (equal); validation (equal); writing – review and editing (equal). **Marley Boyd:** Data curation (equal); formal analysis (equal); investigation (equal); methodology (equal); validation (equal); visualization (lead); writing – review and editing (equal). **Seyed Mahmoudpour:** Methodology (equal); project administration (equal); resources (equal); supervision (equal); writing – original draft (equal); writing – review and editing (equal).

## FUNDING INFORMATION

This study was sponsored by Merck KGaA, Darmstadt, Germany.

## CONFLICT OF INTEREST

David P. Cosgrove reports employment at Compass Oncology, consulting fees from Daiichi Sankyo/Lilly and Merck KGaA, honoraria from Pfizer, and travel meeting support from US Oncology. Emily S. Reese reports employment with EMD Serono. Nicole M. Fulcher reports employment with Ontada. Sarah S. Bobiak reports employment with EMD Serono. Francois‐Xavier Lamy reports employment with Merck KGaA at the time of the study. Arthur Allignol reports employment with Merck KGaA at the time of the study. Marley Boyd reports employment with Ontada at the time of the study. S. Hamidreza Mahmoudpour reports employment with Merck KGaA.

## ETHICAL APPROVAL

Institutional Review Board (IRB) and compliance/privacy approvals were obtained before the initiation of the retrospective research. Exemption status and a waiver of informed consent were granted by The US Oncology IRB (20‐017E). Data were handled in compliance with HIPAA (the US Health Insurance Portability and Accountability Act) and the Health Information Technology for Economic and Clinical Health (HITECH) Act.

## Supporting information


Figure S1

Figure S2

Table S1

## Data Availability

The health data used to support the findings of this study are restricted by the US Oncology Institutional Review Board in order to protect patient privacy. For this reason, data used to support the findings of this study have not been made available.
