# Supplementary figures and images for: Real‐world outcomes among patients with advanced or metastatic biliary tract cancers initiating second‐line treatment
Source: Cancer Med. 2022 Oct 20;12(4):4195–205. doi: 10.1002/cam4.5282 (PMC9972013; doi:10.1002/cam4.5282)

## Supporting Figure 1. Study Attrition

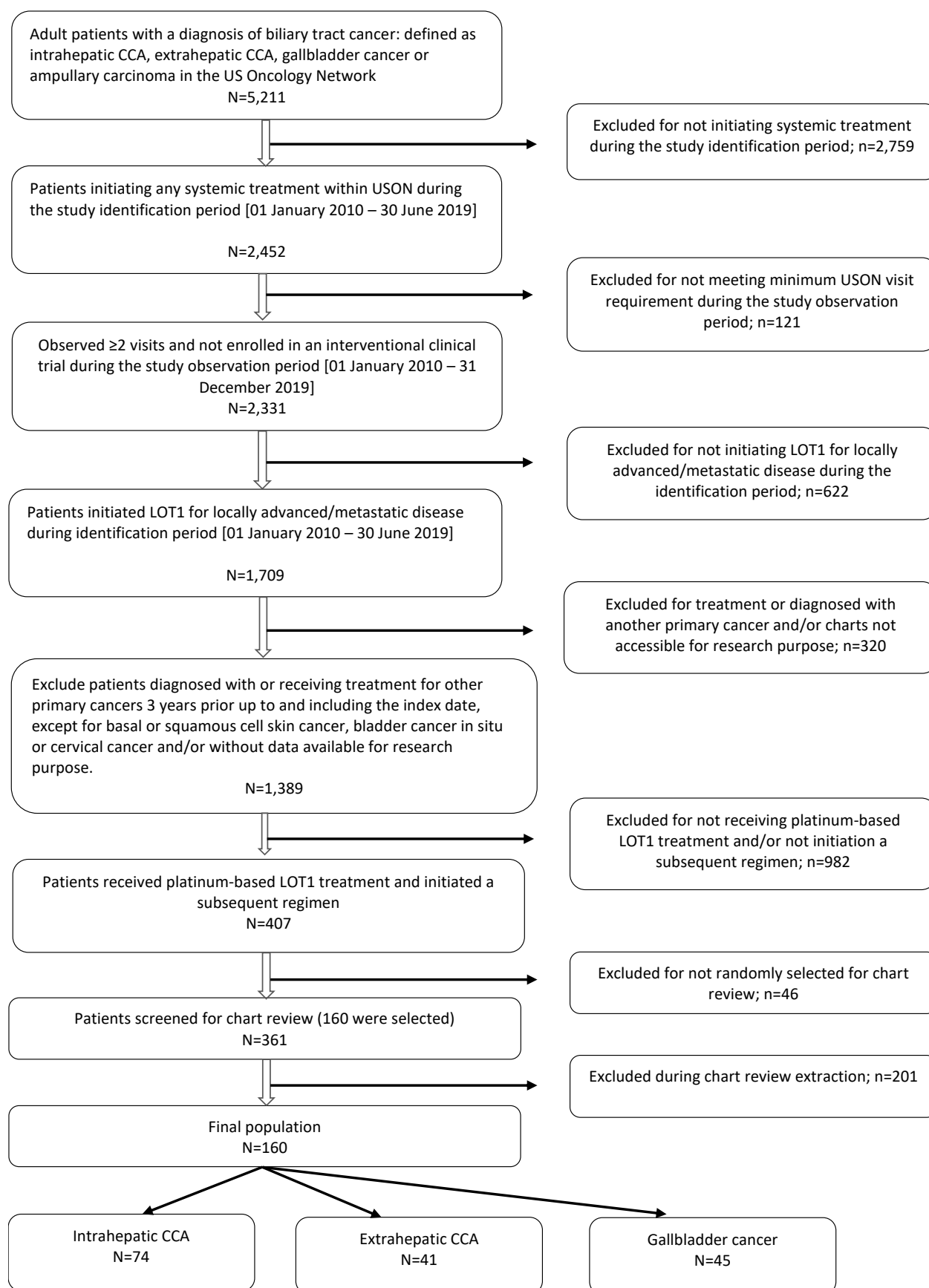

Supplement: Supplementary file 1 — Figure S1 [file CAM4-12-4195-s001.pdf]

Supporting Figure 2. Kaplan Meier Curve and Estimates of Duration of Response

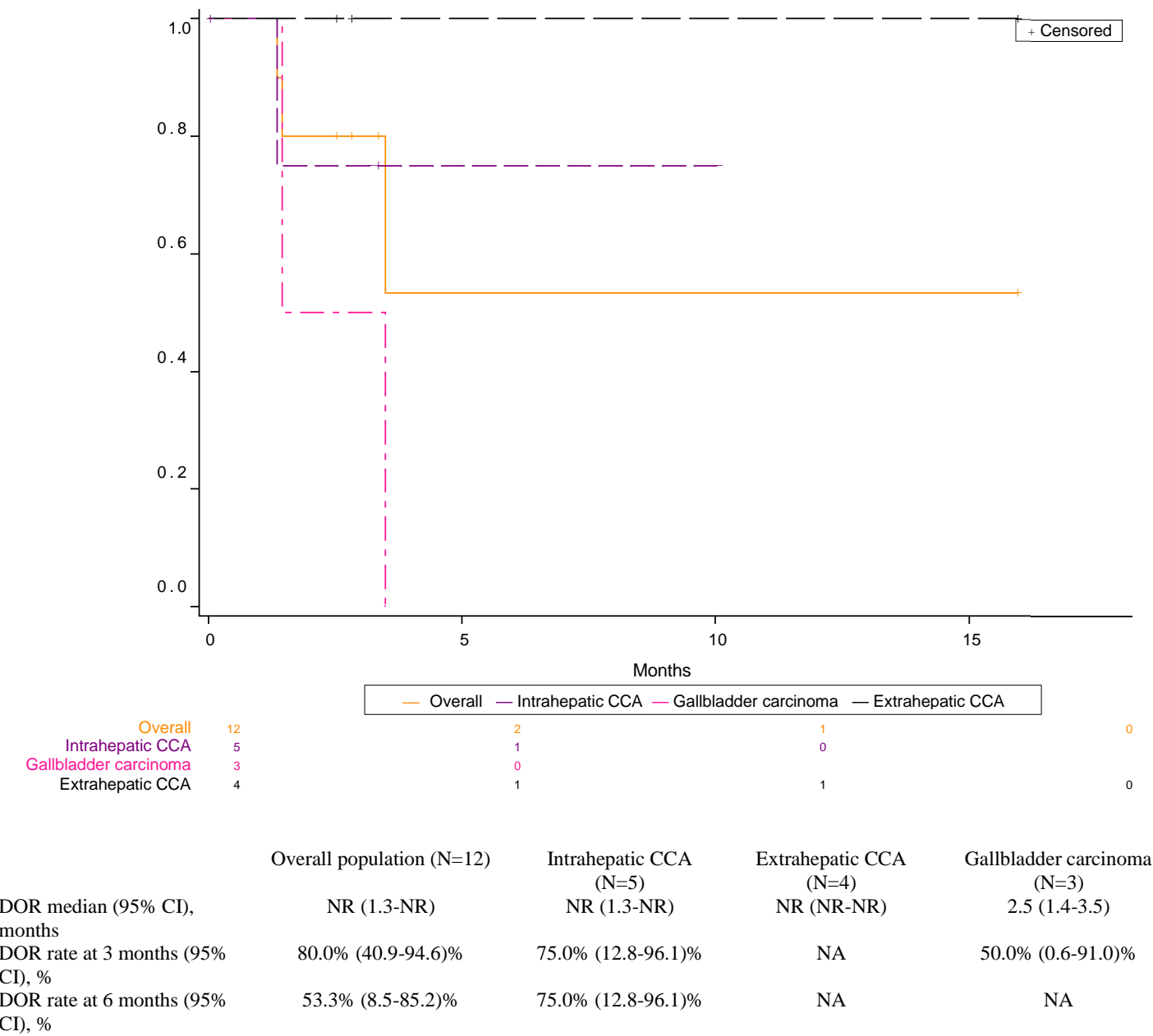

Supplement: Supplementary file 2 — Figure S2 [file CAM4-12-4195-s003.pdf]
