# Supplementary material for: Real‐world outcomes among patients with advanced or metastatic biliary tract cancers initiating second‐line treatment
Source: Cancer Med. 2022 Oct 20;12(4):4195–205. doi: 10.1002/cam4.5282 (PMC9972013; doi:10.1002/cam4.5282)
Supplement: Supplementary file 3 — Table S1 [file CAM4-12-4195-s002.docx]

**Supporting Table 1.** Other Laboratory Measures

|  | | | | **BTC subtype** | | | | |
| --- | --- | --- | --- | --- | --- | --- | --- | --- |
| **Analysis variable** | | **Overall** | | **ICCA** | | **ECCA** | | **Gallbladder** |
| **Patients, n** | | 160 | | 74 | | 41 | | 45 |
| **EGFR result, n (%)** | |  | |  | |  | |  |
| Negative | | 25 (15.6) | | 13 (17.6) | | 3 (7.3) | | 9 (20.0) |
| Positive | | 1 (0.6) | | 0 | | 1 (2.4) | | 0 |
| Not documented | | 134 (83.8) | | 61 (82.4) | | 37 (90.2) | | 36 (80.0) |
| **FGFR1 result, n (%)** | |  | |  | |  | |  |
| Mutation | | 1 (0.6) | | 1 (1.4) | | 0 | | 0 |
| Wild type | | 21 (13.1) | | 10 (13.5) | | 3 (7.3) | | 8 (17.8) |
| Not documented | | 138 (86.3) | | 63 (85.1) | | 38 (92.7) | | 37 (82.2) |
| **FGFR2 result, n (%)** | |  | |  | |  | |  |
| Gene fusion | | 3 (1.9) | | 3 (4.1) | | 0 | | 0 |
| Wild type | | 22 (13.8) | | 10 (13.5) | | 3 (7.3) | | 9 (20.0) |
| Not documented | | 135 (84.4) | | 61 (82.4) | | 38 (92.7) | | 36 (80.0) |
| **IDH-1 result at baseline (2L initiation), n (%)** |  | |  | |  | |  | |
| Wild type | | 2 (1.3) | | 0 | | 0 | | 2 (4.4) |
| Not documented | | 158 (98.8) | | 74 (100.0) | | 41 (100.0) | | 43 (95.6) |

2L, second line; BTC, biliary tract cancer; ECCA, extrahepatic cholangiocarcinoma; EGFR, epidermal growth factor receptor; FGFR1, fibroblast growth factor receptor 1; FGFR2, fibroblast growth factor receptor 2; ICCA, intrahepatic cholangiocarcinoma; IDH-1, isocitrate dehydrogenase 1.
